# Risk Stratification and Surgical De-Escalation in T1 Invasive Lobular Carcinoma: A Retrospective Analysis of Preoperative Axillary Imaging

**DOI:** 10.7759/cureus.117533

**Published:** 2026-10-06

**Authors:** Matthew Fung, Vishnu Karthikeyan, Jenna Doherty, Helen Mathers, Norah Scally

**Affiliations:** 1 Breast Surgery, Craigavon Area Hospital, Portadown, GBR; 2 Breast Surgery, Ulster Hospital, Dundonald, GBR

**Keywords:** axillary ultrasound, breast cancer, invasive lobular carcinoma, preoperative staging, risk stratification, sentinel lymph node biopsy, surgical de-escalation

## Abstract

Background: Invasive lobular carcinoma (ILC) is a common histological subtype of breast cancer, yet preoperative axillary staging remains notoriously challenging due to its diffuse, infiltrative growth pattern. Given the increasing clinical shift toward axillary surgical de-escalation for low-volume disease, precise preoperative evaluation is crucial to guide surgical planning.

Objective: This study aimed to determine the efficacy of preoperative axillary ultrasound and magnetic resonance imaging (MRI), alongside fine needle aspiration cytology (FNAC), in staging ILC and to evaluate the potential for safely omitting sentinel lymph node biopsy (SLNB) in a risk-stratified subgroup.

Methods: A retrospective analysis was conducted on 167 patients diagnosed with operable primary ILC (T1-3, N0-2, M0) treated at a single healthcare trust between February 2020 and August 2024. Electronic care records were reviewed for preoperative imaging and FNAC results, which were then correlated with final postoperative pathology. For nodal classification, isolated tumour cells (ITCs) (pN0(i+)) were treated as node-negative, whereas micrometastases (pN1(mi)) and macrometastases were treated as node-positive. Axillary ultrasound was performed first, with FNAC performed where ultrasound findings required cytological assessment. A negative preoperative axillary assessment was defined as no suspicious nodes on ultrasound and, where FNAC was performed, negative cytology. Nodal status was categorised by burden, and sensitivity, specificity, positive predictive value (PPV), and negative predictive value (NPV) were calculated for each imaging modality.

Results: Of the 167 patients, 58 (34.7%) had node-positive disease. Axillary ultrasound demonstrated an overall sensitivity of 32.8% and a specificity of 100%, while MRI yielded a sensitivity of 29.6% and a specificity of 98.0%. Ultrasound sensitivity varied numerically across tumour-size categories and nodal burden, falling to 0.0% in T1 tumours compared to 40.9% in T2 tumours. Crucially, the prevalence of a high nodal burden (≥3 positive nodes) was extremely rare in T1 tumours (2.4%), in stark contrast to T2 (11.8%) and T3 (45.2%) tumours. The proportion of high nodal burden was low in both age groups.

Conclusions: Neither ultrasound nor MRI can reliably rule out low-volume nodal disease in ILC due to the inherent biological characteristics of the tumour. However, both modalities provide excellent specificity. The high NPV of ultrasound alone in T1 tumours is driven by the fundamentally low prevalence of extensive axillary disease in this subgroup, rather than diagnostic utility. Given that high nodal burden is exceedingly rare in T1 tumours, the hypothesis is that SLNB could potentially be omitted in selected T1 ILC patients. These findings support a risk-stratified approach, suggesting that patients with T1 ILC and a negative preoperative axillary assessment (no suspicious nodes on ultrasound and negative cytology where FNAC was performed) could be considered for surgical de-escalation and omission of SLNB, pending prospective validation.

## Introduction

Breast cancer is the most common cancer affecting women [1]. Preoperative assessment of the axilla forms an important component of staging and surgical planning in patients with invasive breast cancer. Invasive lobular carcinoma (ILC) is the second most common histological subtype of breast cancer, accounting for approximately 10-15% of diagnoses [2].

Despite its frequency, the preoperative staging of the axilla in lobular carcinoma is reported to be less sensitive [3]. ILC nodal metastases are typically more difficult to detect due to the cellular similarity to lymphocytes, alongside the infiltrative pattern throughout the lymph node [4]. This behaviour is associated with the characteristic loss of E-cadherin expression in lobular carcinoma, which results in discohesive cells that infiltrate diffusely rather than forming a cohesive mass [2]. Given the recent shift towards axillary de-escalation in patients with low-volume nodal disease, the accuracy of axillary staging has never been more important, particularly in the planning of subsequent adjuvant systemic therapy [5].

Ongoing clinical trials such as INSEMA (Intergroup-Sentinel-Mamma) [6], BOOG (Borstkanker Onderzoek Groep)-2013-08 [7], NAUTILUS (No Axillary sUrgical Treatment for Lymph node-negative patients after Ultra-Sonography) [8] and SOAPET (Sentinel node biopsy vs. Observation After axillary PET) [9] all continue to look at omission of sentinel lymph node biopsy (SLNB) in clinically node-negative patients undergoing breast-conserving therapy. This study focuses particularly on the subset of lobular carcinoma and the potential for omission of SLNB in a risk-stratified subgroup, since stratification depends on how accurately imaging techniques can evaluate clinically node-negative patients.

The main objective of this study was to evaluate the diagnostic performance of preoperative axillary ultrasound and MRI, alongside FNAC, for detecting axillary nodal metastasis in ILC, with results correlated against final postoperative histopathology. The secondary objectives were to determine the prevalence and distribution of nodal burden across tumour stages, and to assess whether a risk-stratified approach could support omission of SLNB in patients with T1 tumours. Given the retrospective, single-centre design, these objectives are exploratory and hypothesis-generating, and are intended to inform the design of prospective studies rather than to define a change in clinical practice.

## Materials and methods

This was a retrospective cohort analysis conducted on patients diagnosed with primary breast cancer at Craigavon Area Hospital, Southern Health & Social Care Trust, Northern Ireland, between February 2020 and August 2024, identified from a prospectively maintained unit database.

Eligibility criteria and sample size

Inclusion criteria were: (i) female patients aged 18 years or older; (ii) a histologically confirmed primary diagnosis of operable ILC; (iii) clinical tumour stage T1-3, N0-2, M0; and (iv) completion of preoperative axillary staging with ultrasound (with or without MRI).

Exclusion criteria were: (i) non-lobular breast cancer histologies (e.g., purely ductal carcinoma); (ii) history of ipsilateral axillary surgery; (iii) presence of distant metastatic disease (M1); (iv) incomplete electronic medical records or missing postoperative pathology data; and (v) revision of histological subtype to non-lobular carcinoma on final post-resection pathology.

Initially, 168 patients who met the preliminary inclusion criteria were identified. One patient was excluded due to missing final postoperative pathological data. This resulted in a final analytical study population of 167 patients.

Data collection and classification

Electronic care records were reviewed by two independent reviewers (MF and VK) to extract data on preoperative staging modalities, including axillary ultrasound, MRI, and FNAC. Where discrepancies arose between the two independent reviewers, cases were reviewed jointly and resolved by consensus, with adjudication by a third reviewer (NS) where agreement could not be reached. These preoperative findings were then correlated with the final postoperative pathology results to determine the diagnostic accuracy of each imaging modality.

As an NHS Breast Screening Programme centre, axillary FNAC was performed according to local protocol on any lymph node demonstrating a cortical thickness of ≥3 mm or loss of the fatty hilum. Where FNAC was insufficient (C1; inadequate sample), repeat FNAC was performed and, where necessary, core biopsy was undertaken to confirm a benign diagnosis.

All patients underwent preoperative axillary ultrasound. FNAC was performed where ultrasound findings required cytological assessment. Preoperative axillary status was classified as negative if ultrasound showed no suspicious nodes and, where FNAC was performed, cytology was negative; positive cytology was classified as positive preoperative axillary status. Thus, "negative preoperative axillary assessment" refers to no suspicious nodes on ultrasound and negative cytology where FNAC was performed.

Tumour size was classified according to the standardized TNM staging system for breast cancer (8th edition, American Joint Committee on Cancer (AJCC)) [10]. Briefly, primary tumour (pT) sizes were categorised as T1 (≤ 20 mm), T2 (> 20 mm to ≤ 50 mm), or T3 (> 50 mm). Nodal status was categorised as low burden (1-2 positive nodes) or high burden (≥3 positive nodes with macrometastatic or micrometastatic disease). Micrometastases were considered node-positive in this study, whereas ITCs were classified as node-negative. No distinction was made between unifocal and multifocal disease in the assessment of nodal status.

Statistical analysis

Data collection and management were performed using Microsoft Excel (Microsoft Corporation, Redmond, Washington, United States). Statistical analysis was conducted using MedCalc’s online diagnostic test calculator (MedCalc Software Ltd, Ostend, Belgium; available at: https://www.medcalc.org/calc/diagnostic_test.php). Descriptive statistics were used to summarise patient demographics and the biological prevalence of nodal burden across tumour stages. The diagnostic performance of each preoperative imaging modality was evaluated by calculating the sensitivity, specificity, positive predictive value (PPV), and negative predictive value (NPV). Results were presented alongside their respective 95% confidence intervals (CIs) to ascertain statistical reliability.

## Results

A total of 1532 patients, with a primary breast cancer diagnosis, were identified from the unit database between February 2020 and August 2024. Of these, 168 patients were identified with a diagnosis of ILC (11%). Demographic analysis demonstrated a mean age of 60.6 years (range 35-83 years) across the final analysed cohort of 167 patients (Table 1).

**Table 1:** Patient demographics and clinical characteristics (N = 167) pT: pathological tumour stage; pN: pathological nodal burden; USS: ultrasound; MRI: magnetic resonance imaging; FNAC: fine needle aspiration cytology

Characteristic		Value
Age (years)	Mean (range)	60.6 (35-83)
	Median	61
Pathological tumour Stage (pT), n (%)	T1	85 (50.9%)
	T2	51 (30.5%)
	T3	31 (18.6%)
Pathological Nodal Burden (pN), n (%)	Negative (0 nodes)	109 (65.3%)
	Low Burden (1–2 nodes)	36 (21.6%)
	High Burden (≥3 nodes)	22 (13.2%)
Preoperative Staging Modality, n (%)	Ultrasound (USS)	167 (100%)
	MRI	154 (92.2%)
	FNAC	33 (19.8%)

Retrospective analysis of the 167 patients determined that all patients had an axillary ultrasound as part of their preoperative staging, with subsequent FNAC to further clarify suspicious lesions, and 154 (92.2%) had a preoperative MRI. Of the patients who did not have an MRI, there was documented rationale for omission, including claustrophobia, physical disability prohibiting MRI, and elevated BMI. A total of 58 patients (34.7%) had node-positive disease on final postoperative histopathology. Of those who were node-positive, 36 (62.1%) were classified as low burden, and 22 (37.9%) had high burden disease. 

Preoperative MRI yielded a sensitivity of 29.6%, a specificity of 98.0%, a PPV of 88.9%, and a NPV of 72.1% (Table 2, Figure 1). In comparison, axillary ultrasound demonstrated a higher overall sensitivity of 32.8% and a marginally higher NPV of 73.7%, alongside 100% specificity and 100% PPV. When evaluating ultrasound performance based on the extent of metastasis, sensitivity was 13.9% for low tumour burden (1-2 nodes) and increased to 63.6% for high tumour burden (≥3 nodes). Notably, the prevalence of this high nodal burden was only 2.4% in T1 tumours, compared with 11.8% in T2 tumours and 45.2% in T3 tumours (Figure 2).

**Table 2:** Diagnostic performance of imaging modalities ^a^ PPV could not be calculated for the T1 subgroup, as axillary ultrasound detected no true-positive cases in this group (sensitivity 0.0%); PPV is therefore reported as not applicable ("–"). CI: confidence interval; PPV: positive predictive value; NPV: negative predictive value; FNAC: fine needle aspiration cytology; MRI: magnetic resonance imaging

Imaging Modality		Sensitivity (%) (95% CI)	Specificity (%) (95% CI)	PPV (%) (95% CI)	NPV (%) (95% CI)
Axillary Ultrasound	Overall	32.8 (21.0 to 46.3)	100.0 (96.7 to 100.0)	100.0 (82.4 to 100.0)	73.7 (70.0 to 77.0)
	Low Burden (1-2 nodes)	13.9 (4.7 to 29.5)	100.0 (96.7 to 100.0)	100.0 (47.8 to 100.0)	77.9 (75.5 to 80.0)
	High Burden (≥3 nodes)	63.6 (40.7 to 82.8)	100.0 (96.7 to 100.0)	100.0 (76.8 to 100.0)	93.2 (88.7 to 96.0)
	T1	0.0 (0.0 to 21.8)	100.0 (94.9 to 100.0)	-^a^	82.4 (82.4 to 82.4)
	T2	40.9 (20.7 to 63.7)	100.0 (88.1 to 100.0)	100.0 (66.4 to 100.0)	69.1 (61.2 to 76.0)
	T3	47.6 (25.7 to 70.2)	100.0 (69.1 to 100.0)	100.0 (69.2 to 100.0)	47.6 (37.7 to 57.8)
Preoperative MRI		29.6 (18.0 to 43.6)	98.0 (93.0 to 99.8)	88.9 (65.7 to 97.1)	72.1 (68.4 to 75.5)
FNAC		81.8 (59.7 to 94.8)	84.6 (54.6 to 98.1)	90.0 (71.2 to 97.0)	73.3 (52.4 to 87.3)

**Figure 1:**
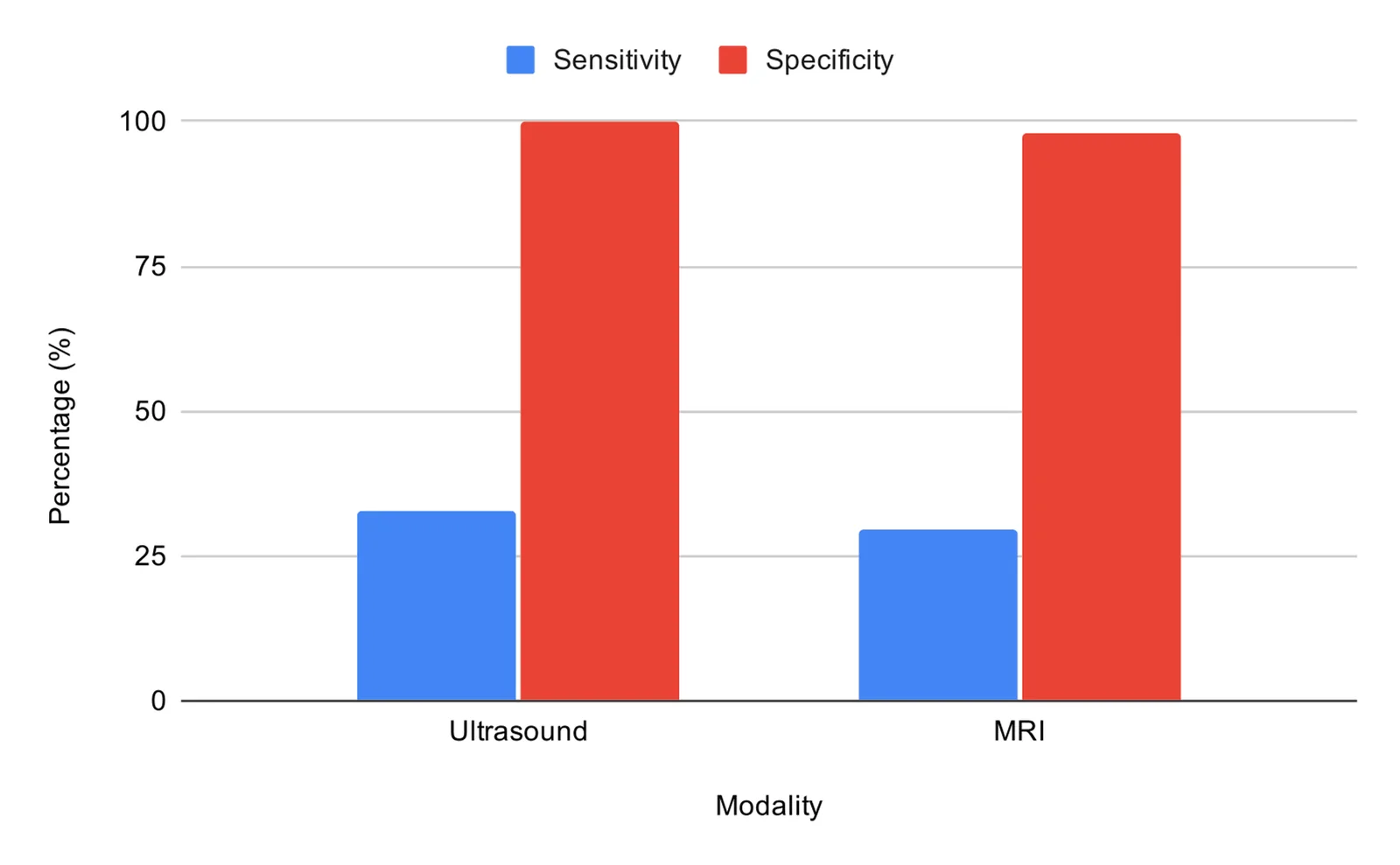
Comparison of sensitivity and specificity of preoperative axillary ultrasound versus preoperative MRI in detecting nodal disease MRI: magnetic resonance imaging.

**Figure 2:**
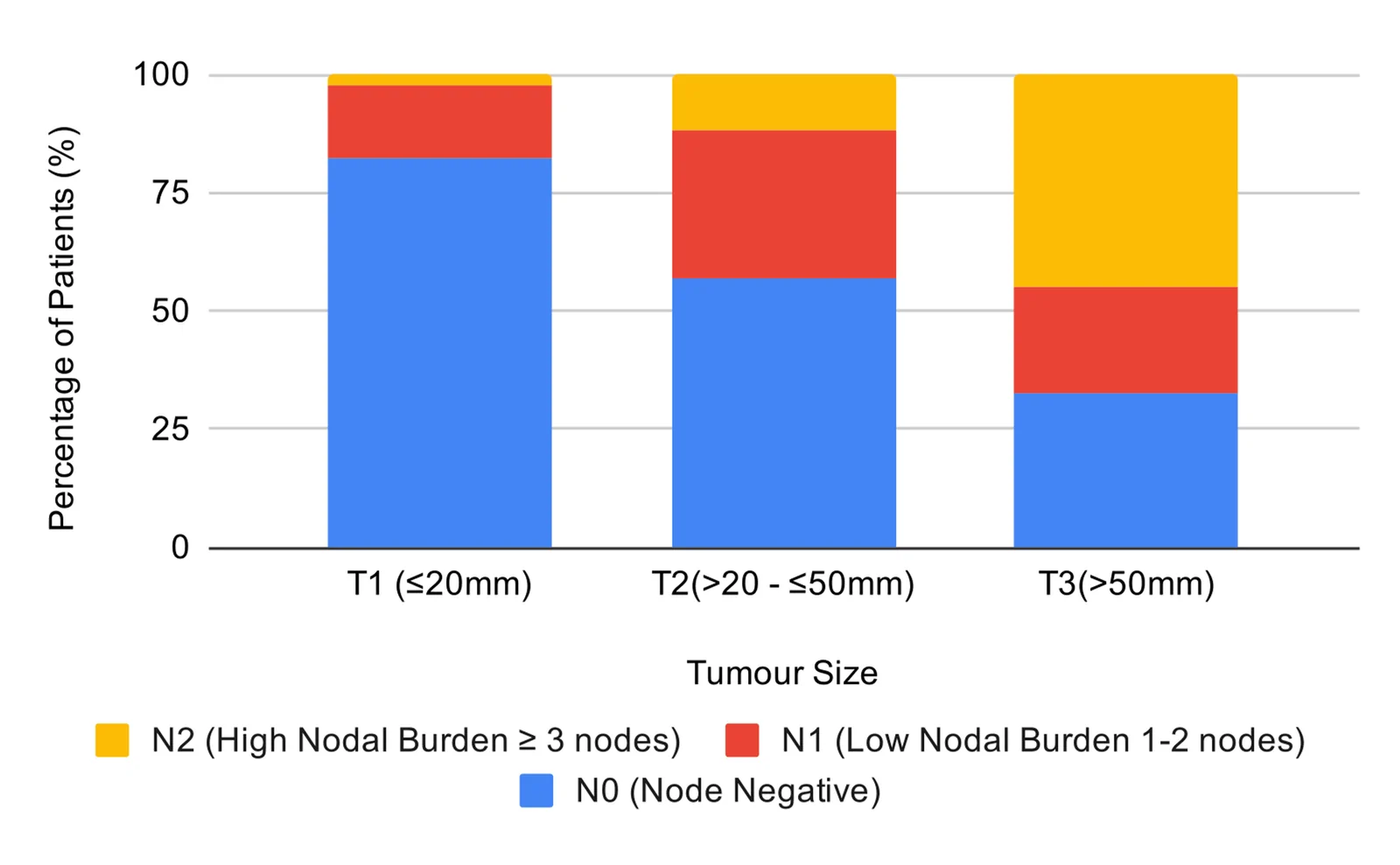
Prevalence of nodal burden between T1, T2 and T3 tumours T1: Primary tumour ≤ 20 mm; T2: Primary tumour > 20 mm to ≤ 50 mm; T3: Primary tumour > 50 mm.

When stratified by age, the diagnostic performance of both modalities remained poor across both cohorts. In patients aged ≤60 years, ultrasound demonstrated a sensitivity of 35.7% (95% CI 18.6-55.9) and a specificity of 100.0% (95% CI 94.0-100.0), compared with a sensitivity of 31.0% (95% CI 15.3-50.8) and specificity of 98.0% (95% CI 89.4-100.0) in those aged >60 years. Similarly, MRI demonstrated a sensitivity of 26.9% (95% CI 11.6-47.8) and 32.1% (95% CI 15.9-52.4) in the ≤60 and >60 age cohorts respectively. The prevalence of high-burden nodal disease in T1 tumours remained exceptionally low in both groups (2.3% in patients aged ≤60 years and 2.4% in those aged >60 years), suggesting that the favourable biology of T1 ILC is independent of patient age (Table 3). 

**Table 3:** Demographics, pathological nodal status, and diagnostic imaging performance stratified by age cohort T1: Primary tumour ≤ 20 mm; T2: Primary tumour > 20 mm to ≤ 50 mm; T3: Primary tumour > 50 mm. High nodal burden was defined as ≥3 positive nodes. Age cohorts were defined as ≤60 years and >60 years. CI: confidence interval; PPV: positive predictive value; NPV: negative predictive value; MRI: magnetic resonance imaging

Characteristic		Age ≤ 60 Years (n = 88)	Age > 60 Years (n = 79)
Primary Tumour Size, n (%)	T1	43 (48.9%)	42 (53.2%)
	T2	31 (35.2%)	20 (25.3%)
	T3	14 (15.9%)	17 (21.5%)
	T1 with nodal positive disease	6 (14.0%)	8 (19.0%)
	T1 with high nodal disease	1 (2.3%)	1 (2.4%)
Pathological Nodal Status, n (%)	Node positive	30 (34.1%)	28 (35.4%)
	High nodal burden (≥3 nodes)	11 (12.5%)	11 (13.9 %)
Ultrasound Performance (%) / (95% CI)	Sensitivity	35.7 (18.6 to 55.9)	31.0 (15.3 to 50.8)
	Specificity	100.0 (94.0 to 100.0)	98.0 (89.4 to 100.0)
	Positive Predictive Value (PPV)	100.0 (69.2 to 100.0)	90.0 (54.6 to 98.5)
	Negative Predictive Value (NPV)	76.9 (71.7 to 81.5)	71.0 (65.7 to 75.8)
Preoperative MRI Performance (%) / (95% CI)	Sensitivity	26.9 (11.6 to 47.8)	32.1 (15.9 to 52.4)
	Specificity	96.2 (87.0 to 99.5)	100.0 (92.6 to 100.0)
	Positive Predictive Value (PPV)	77.8 (43.9 to 94.0)	100.0 (66.4 to 100.0)
	Negative Predictive Value (NPV)	72.9 (67.9 to 77.3)	71.6 (66.2 to 76.5)

## Discussion

The preoperative axillary staging of ILC remains notoriously challenging. Our retrospective study sought to evaluate the efficacy of current imaging modalities in this specific cohort. The data highlighted that while axillary ultrasound marginally outperformed MRI in overall sensitivity (32.8% vs. 29.6%), neither modality proved reliable in safely ruling out nodal disease. This finding is concordant with current research and is reflected in existing radiological guidelines [11,12]. However, both modalities demonstrated excellent specificity (≥98%), confirming the overarching clinical theme for ILC: positive imaging reliably "rules in" nodal disease, but negative imaging cannot safely "rule out" the presence of low-volume metastasis.

The diagnostic limitations of imaging are rooted in the unique biology of lobular breast cancer. Unlike other breast cancers, ILC typically exhibits a diffuse, infiltrative growth pattern that fails to form a distinct mass or cause significant tissue distortion [13]. Lack of a distinct mass makes primary breast lesions difficult to detect on standard mammography and similarly masks metastatic deposits within the axillary lymph nodes. This subtle growth pattern explains why low-volume axillary disease is so frequently missed on imaging, with ultrasound demonstrating only a 13.9% sensitivity for detecting one to two positive nodes. Consequently, our data show that imaging accuracy relies heavily on tumour size and nodal burden, with ultrasound sensitivity dropping to just 0.0% in T1 tumours compared to 40.9% in T2 tumours. This is likely secondary to the low prevalence of nodal metastasis within the T1 cohort and the correspondingly small sample size of true positive nodes.

Despite the overall low sensitivity of imaging, dividing the cohort by tumour size demonstrates a potential metric for risk stratification. Our data demonstrated a significant contrast in the prevalence of axillary disease between T1 and T2 tumours. Only 17.6% of T1 tumours presented with any positive nodal disease, and crucially, a mere 2.4% exhibited a high nodal burden (≥3 nodes). Conversely, T2 tumours showed a 43.2% rate of positive nodal disease, with 11.8% harbouring a high nodal burden. While ultrasound performance improves as nodal burden increases (63.6% of high-burden disease vs. 13.9% of low-burden), the critical finding lies in the NPV. Given the inherently low prevalence of extensive axillary disease in smaller tumours, the high NPV (82.4%) of a negative ultrasound in T1 tumours is driven almost entirely by a low pre-test probability, rather than the diagnostic utility of the scan itself. In fact, with ultrasound demonstrating a 0.0% sensitivity for detecting positive nodes in our T1 cohort, the imaging effectively adds no diagnostic value in this specific subgroup; a negative scan is virtually guaranteed regardless of true nodal status. Therefore, the clinical reassurance that a patient is highly unlikely to harbour ≥3 positive nodes stems from the low prevalence of extensive nodal disease observed in T1 tumours, rather than the negative radiological finding. The combination of T1 tumour stage and a clinically negative axilla was associated with a low prevalence of high nodal burden in this cohort.

Current guidelines from the National Institute for Health and Care Excellence (NICE) (United Kingdom) [14] and American Society of Clinical Oncology (United States) [15] provide a clear framework for de-escalating axillary surgery, heavily influenced by the American College of Surgeons Oncology Group (ACOSOG) Z0011 trial [16]. These guidelines advise omitting axillary lymph node clearance (ALNC) for patients undergoing breast-conserving surgery who are clinically node-negative and have two or fewer positive nodes identified on SLNB, provided they will receive whole-breast radiotherapy. The rationale is that the severe morbidity risks of ALNC outweigh the benefits, with no significant impact on long-term regional recurrence or overall survival [14].

Importantly, age stratification in our cohort demonstrated that the low prevalence of extensive nodal disease observed in T1 tumours remains consistent regardless of the patient's age (Table 3). While the preoperative sensitivity of both ultrasound and MRI remained universally low across the ≤60 and >60 age cohorts, the incidence of high-burden nodal disease in T1 tumours remained exceptionally low across both demographic groups. These findings are conceptually aligned with the SOUND (Sentinel node versus Observation after axillary ultra-soUND) trial [17], which evaluated omission of sentinel lymph node biopsy in selected patients with small breast cancers and a negative preoperative axillary ultrasound. Therefore, if a patient with a T1 tumour and a negative scan undergoes an SLNB, the procedure is highly likely to reveal either node-negative or low-burden disease, neither of which mandates an ALNC under current guidelines [14].

It is important to acknowledge, however, that omitting SLNB is not equivalent to omitting ALNC. While ACOSOG Z0011 demonstrates that low-burden axillary disease can be safely managed without clearance, those trials relied on sentinel node status to identify the patients in whom clearance could be avoided [16]. Omitting SLNB entirely therefore removes not only the surgical procedure but also the nodal information on which some adjuvant decisions may depend. Genomic testing may still be indicated in T1 lobular cancers with fewer than three involved nodes, and knowledge of nodal status can influence both the decision to request such assays and the interpretation of their results. In postmenopausal women with a T1 tumour and one to two positive nodes, genomic testing may be used to determine whether adjuvant chemotherapy is warranted; however, where a patient would not be considered a candidate for chemotherapy on the basis of age, comorbidity or patient preference, the finding of low-burden nodal disease is unlikely to alter management. In such cases, omission of SLNB may be a reasonable consideration, since the nodal information would not change the treatment decision.

Under current NICE guidance, eligibility for adjuvant abemaciclib in hormone receptor-positive, human epidermal growth factor receptor 2 (HER2)-negative early breast cancer is restricted to high-risk disease, defined as ≥4 positive axillary lymph nodes, or one to three positive nodes alongside grade 3 disease or a primary tumour size of at least 5 cm [18]. Omission of SLNB in T1 lobular cancer would therefore be unlikely to alter eligibility for CDK4/6 inhibition in this cohort, since only 2.4% of T1 tumours harboured high-burden disease. The impact of omission is thus likely to be confined to a small minority of patients in whom the finding of low-burden nodal disease would meaningfully change the recommendation for chemotherapy or genomic testing. Our findings therefore support a risk-stratified, shared decision-making approach in which omission of SLNB may be considered in T1 ILC with a negative preoperative axillary assessment, particularly where avoidance of axillary surgical morbidity is considered clinically desirable.

Given this minimal clinical benefit, routinely subjecting T1 patients with negative imaging to surgical axillary staging should be re-examined. Axillary surgery, while SLNB is less invasive than ALNC, still carries tangible long-term morbidity. Complications such as axillary paraesthesia (affecting up to 11% of patients) and chronic lymphoedema (5% of patients) frequently impact a patient's quality of life and activities of daily living [19]. Our findings suggest adopting a risk-stratified approach could potentially omit axillary surgery for patients with T1 and a negative ultrasound (Figure 3). This aligns closely with recent de-escalation trials (such as SOUND [17] and INSEMA [6]), which advocate for the safety of omitting SLNB in small breast cancers with negative axillary imaging. Importantly, this de-escalation is safely restricted to smaller lesions; the observed 11.8% prevalence of high nodal burden in T2 tumours provides less support for extending this proposed de-escalation strategy to T2 disease.

**Figure 3:**
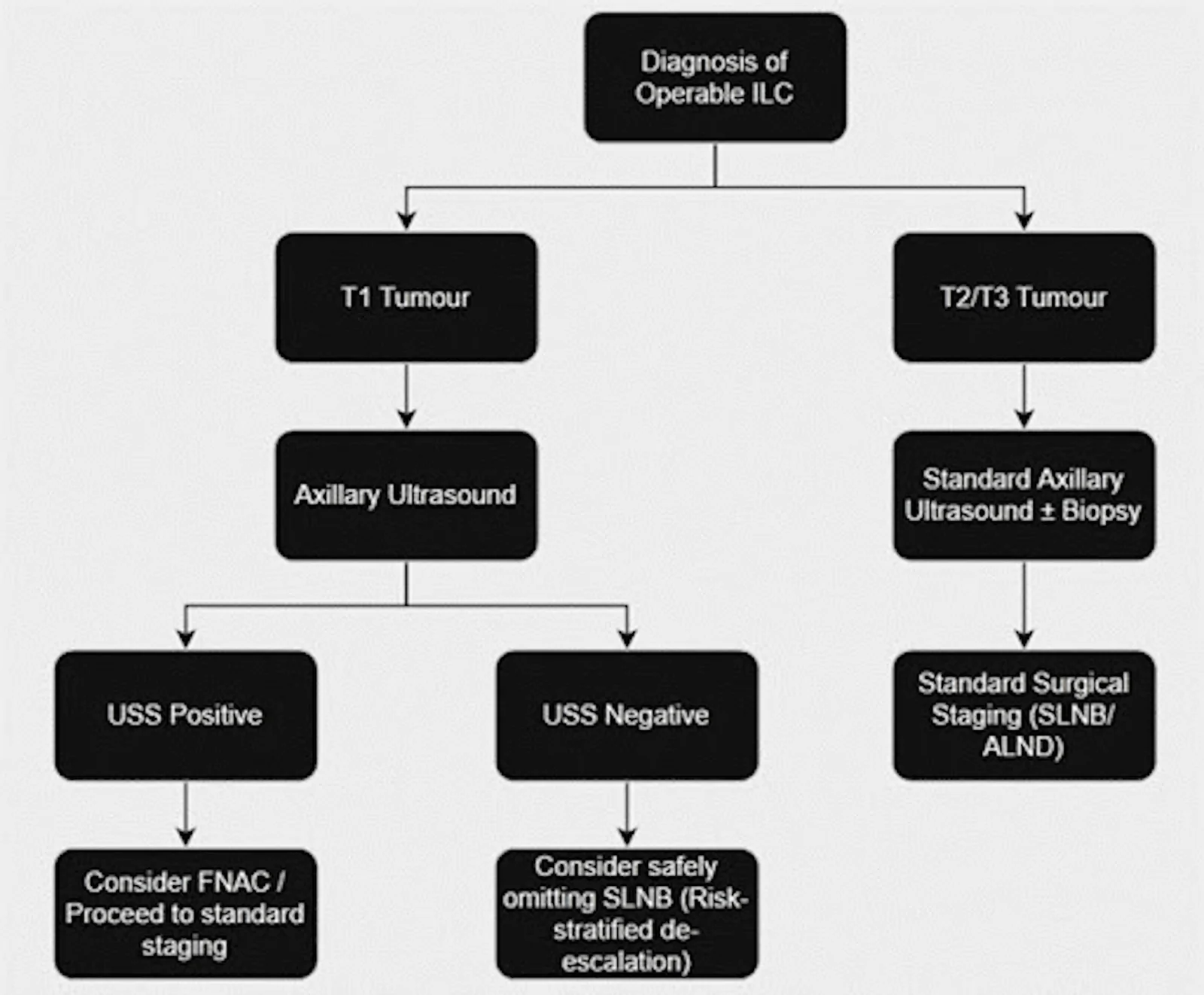
Proposed risk stratification pathway T1: primary tumour ≤ 20 mm; T2: primary tumour > 20 mm to ≤ 50 mm; T3: primary tumour > 50 mm ILC: invasive lobular carcinoma; USS: ultrasound; FNAC: fine needle aspiration cytology; SLNB: sentinel lymph node biopsy; ALND: axillary lymph node dissection

The present study is subject to several limitations inherent to its retrospective, single-unit design. As a single-centre study in Northern Ireland, there is an inherent risk of selection bias, and the results may not be entirely generalisable to healthcare systems utilising different imaging protocols or facing varied surgical volumes. Furthermore, the overall low sensitivity of ultrasound (32.8%) and MRI (29.6%) for axillary staging in ILC remains a recognised limitation of the modalities themselves. Axillary ultrasound interpretation is also operator-dependent, and longer follow-up is required before omission of SLNB in T1 ILC could be adopted in practice.

Several limitations specific to the T1 subgroup warrant emphasis. Ultrasound demonstrated 0.0% sensitivity in T1 tumours, meaning all node-positive T1 patients were classified as node-negative preoperatively. While high-burden disease was rare in this subgroup (2.4%), a further proportion had low-burden nodal involvement that would not have been detected. Although low-burden disease does not mandate ALNC under current guidelines [14], nodal status may still influence adjuvant systemic therapy decisions in selected patients, and omission of SLNB therefore risks forgoing information that could alter management in a minority. The T1 node-positive subgroup was also small, with few events underpinning the diagnostic metrics and correspondingly wide confidence intervals; these subgroup analyses are therefore underpowered and should be regarded as exploratory and hypothesis-generating rather than definitive. Nevertheless, the low risk of high nodal burden in T1 tumours with a negative ultrasound supports the development of a risk-stratified hypothesis worthy of prospective evaluation. To validate this risk stratification and justify a formal shift in clinical guidelines, multi-centre trials specifically powered to assess the omission of axillary staging in early-stage ILC patients are essential.

## Conclusions

The preoperative radiological staging of the axilla in ILC remains challenging due to the tumour's inherent infiltrative growth pattern. Our findings confirm that ultrasound marginally outperforms MRI as an imaging modality for detecting and staging ILC; however, neither can safely rule out low-volume nodal disease. Conversely, both ultrasound and MRI demonstrate excellent specificity for reliably identifying positive nodes. Crucially, this study demonstrates that high nodal burden is exceedingly rare in T1 tumours. As such, the clinical reassurance that extensive nodal disease is absent stems primarily from the favourable biology of T1 ILC, rather than the negative predictive value of the ultrasound itself.

Current guidelines recommend surgical axillary staging for patients with clinically node-negative invasive breast cancer who do not meet specified criteria for omission. Our findings suggest that this approach may merit re-examination in this specific subgroup. Aligning with ongoing research into surgical de-escalation, our data support a risk-stratified approach in which omission of SLNB could be considered for T1 ILC with a negative preoperative axillary assessment, within a shared decision-making framework. Given the retrospective, single-centre design and the small T1 node-positive subgroup, these findings should be regarded as exploratory and hypothesis-generating, and require prospective validation before any change in clinical practice.
